# Utilization of Molecular Dynamics Simulation Coupled with Experimental Assays to Optimize Biocompatibility of an Electrospun PCL/PVA Scaffold

**DOI:** 10.1371/journal.pone.0169451

**Published:** 2017-01-24

**Authors:** Morteza Sarmadi, Amir Shamloo, Mina Mohseni

**Affiliations:** Department of Mechanical Engineering, Sharif University of Technology, Tehran, Iran; University of Leeds, UNITED KINGDOM

## Abstract

The main focus of this study is to address the possibility of using molecular dynamics (MD) simulation, as a computational framework, coupled with experimental assays, to optimize composite structures of a particular electrospun scaffold. To this aim, first, MD simulations were performed to obtain an initial theoretical insight into the capability of heterogeneous surfaces for protein adsorption. The surfaces were composed of six different blends of PVA (polyvinyl alcohol) and PCL (polycaprolactone) with completely unlike hydrophobicity. Next, MTT assay was performed on the electrospun scaffolds made from the same percentages of polymers as in MD models to gain an understanding of the correlation between protein adsorption on the composite surfaces and their capability for cell proliferation. To perform simulations, two ECM (extracellular matrix) protein fragments, namely, collagen type I and fibronectin, two essential proteins for initial cell attachment and eventual cell proliferation, were considered. To evaluate the strength of protein adsorption, adhesion energy and final conformations of proteins were studied. For MTT analysis, different blends of PCL/PVA electrospun scaffolds were prepared, on which endothelial cells were cultured for one week. Theoretical results indicated that the samples with more than 50% of PCL significantly represented stronger protein adsorption. In agreement with simulation results, experimental analysis also demonstrated that the more hydrophobic the surface became, the better initial cell attachment and cell proliferation could be achieved, which was particularly better observed in samples with more than 70% of PCL.

## Introduction

As the modern tissue engineering is rapidly going toward utilizing composite biocompatible scaffolds, demands for obtaining the best biocompatibility, providing optimum cell adhesion and proliferation, become more serious. Polymeric composite scaffolds, composed of different hydrophobic and hydrophilic polymers are widely prevalent nowadays due to providing a better control over scaffold characteristics [1–3]. However, as utilizing different percentages of each type of polymer leads to different scaffold characteristics, finding the appropriate optimum percentages of polymers, providing the best cell attachment and biocompatibility, remains a challenging issue in modern tissue engineering. There are experimental assays to examine the capability of cell adhesion and proliferation on scaffolds. However, they may not be simple, especially, for a large number of samples, since the fabrication process and biocompatibility assay should be performed for all of the proposed structures, which can be both expensive and time-consuming. Therefore, a computational approach providing a good estimate of the capability of cell attachment on the scaffolds, can be considered as a significant tool, narrowing down the number of samples, thus facilitating the optimization process. Particularly, this approach can be useful for composite scaffolds composed of several types of polymers, or novel types of polymeric biomaterials.

It is generally believed that success or failure of scaffolds is highly dependent on their potential for specific adsorption of target proteins, particularly those comprising the ECM, since they represent a leading role in initial cell attachment and cell proliferation [4–6]. Additionally, undesirable non-specific blood protein adsorption in medical implants may even increase the risk of blood clotting. A desirable computational approach, capable of quantifying the protein adsorption strength, not only should accurately accommodate the adsorption process, but also should predict the capability of cell attachment on the substrate [7]. In recent years, molecular dynamics (MD) simulation has been emerged as an efficient method for simulation of different biotechnology related phenomena in nanoscales [8], since it accurately models fully-atomistic interactions between biomolecules and surfaces in nanoscales.

MD simulation has been widely employed in the literature for a variety of surfaces including structured surfaces and carbon nanostructures [9–15], but there are only a few studies for polymeric amorphous surfaces [16, 17]. In all of these researches, interaction energies between protein and surface have been studied as the main criterion for assessment of protein adsorption strength. These interactions are observed to change based on a number of factors including protein conformational rearrangements and deformations [9,13], protein size [17] and hydrophobicity [12,13,17], and most importantly surface hydrophobicity and functionalization [12–17]. Lack of extensive studies on the simulation of protein adsorption on polymeric amorphous surfaces can be attributed to highly complex rearrangements and denaturation of proteins in the physisorption process relative to the surfaces [18,19]. MD simulation of the protein adsorption on the heterogeneous surfaces composed of several polymers is highly applicable in tissue engineering, since the composite scaffold can be optimized and better designed, thus, the number of samples for experimental biocompatibility assay decreases.

In this study, first, we use MD simulations to model the protein adsorption on heterogonous polymeric surfaces composed of different percentages of PCL and PVA in a water-like medium. In this regard, two paramount ECM proteins with similar size but unlike secondary structures, including fibronectin and collagen type I are considered for MD simulations. Afterwards, an experimental cell viability assay, MTT assay, is performed for the electrospun scaffolds with the same percentages of PCL and PVA as in MD simulations. Subsequently, we compare the results of protein adsorption with those of cell proliferation for all samples. This comparison can provide insight into the biocompatibility of surfaces from two different points of view including computational approach of MD simulations in nanoscales and experimental MTT assay in macro (cellular) scales.

The heterogeneous composite surfaces are composed of two popular polymers, namely, PCL and PVA extensively used in tissue engineering, and drug delivery [20–25]. In contrast to PCL which is a hydrophobic polymer, PVA is extremely hydrophilic, hence, their blends with various percentages of polymers represent different physicomechanical characteristics. In this study, six surfaces including four heterogonous PCL/PVA scaffolds in addition to two pure PCL and PVA surfaces with nanoscale dimensions are generated for MD simulation to quantify the adsorption of the proteins. The adhesion energy and final confirmation of proteins are studied to evaluate the interaction of proteins and surfaces in nanoscales. MTT assay is also performed to compare the biocompatibility of electrospun PCL/PVA scaffolds and their capability in ECM protein adsorption.

Our hypothesis is that results of MD simulations can demonstrate a good estimate of the overall trend in biocompatibility of different scaffolds. This study suggests to initially optimize composition of polymeric scaffolds using protein adsorption obtained from MD results, leading to optimum design of the scaffold, which can later eliminate the samples with low protein adsorption. Subsequently, one can carry out the experimental *in vitro* and *in vivo* studies on the fewer remaining samples. This study is the first to address a parallel computational-experimental approach toward optimizing a heterogeneous polymeric PCL/PVA scaffold for the best biocompatibility. Results of this study can give insight into how effective MD simulations can be for optimum design of biocompatible scaffolds, reducing the need for performing extensive experimental assays.

## Materials and Methods

### MD Simulation parameters

Classical MD simulations are employed to simulate adsorption of proteins onto amorphous biomaterials. In this paper, all simulations have been carried out using Material Studio (MS) software considering Dreiding [26] as the considered force-field. Dreiding is a generic forcefield that has been extensively employed for molecular modeling of proteins and polymers [26–28]. The non-bonded interactions in Dreiding can be broken down into van der Waals (E_vdW_), Columbic representation of electrostatic interactions (E_electrostatic_), as well as explicit hydrogen bonds (E_hb_):

$$E_{non-bonded}=E_{vdW}+E_{electrostatic}+E_{hb}$$ (1)

also *E*_*vdW*_ is calculated by:

$$E_{vdW}(r_{ij})=\left\{ \begin{array}{c} 0,r_{ij}\geq r_{cut}\\ 4\varepsilon_{ij}\left[{\left(\frac{\sigma_{ij}}{r_{ij}}\right)}^{12}-{\left(\frac{\sigma_{ij}}{r_{ij}}\right)}^{6}\right] \end{array}\right.,r_{ij}<r_{cut}$$ (2)

where *r*_*ij*_ denotes the separation distance, *ε*_*ij*_ is the equilibrium energy, *σ*_*ij*_ is the equilibrium distance between two atoms of *i* and *j*, while *r*_*cut*_ is the cut-off distance beyond which no interaction is considered. These values for each pair of similar or different atoms are specified based on the particular force field. *E*_*electrostatic*_ is also equal to:

$$E_{electrostatic}=\frac{q_{i}q_{j}}{\varepsilon_{eff}r_{ij}}$$ (3)

where *q*_*i*_ and *q*_*j*_ denote atomic charges on the *i*th and *j*th atoms, *r*_*ij*_ represents the distance between these atoms, and *ϵ*_*eff*_ is the dielectric constant. Finally, the hydrogen bond interactions are also calculated as:

$$E_{hb}=D_{hb}[5{\left(R_{hb}/R_{DA}\right)}^{12}-6{\left(R_{hb}/R_{DA}\right)}^{10}]\cos^{4}(\theta_{DHA})$$ (4)

where θ_DHA_ is the bond angle between the hydrogen donor (D), the hydrogen (H), and the hydrogen acceptor (A), and R_DA_ is the distance in Å, between the interacting donor and acceptor atoms, also D_hb_ and R_hb_ are the hydrogen bond constants that can be found in [26]. More explanations regarding the above-mentioned terms can be found in [26].

#### Collagen type I

**Fig 1:**
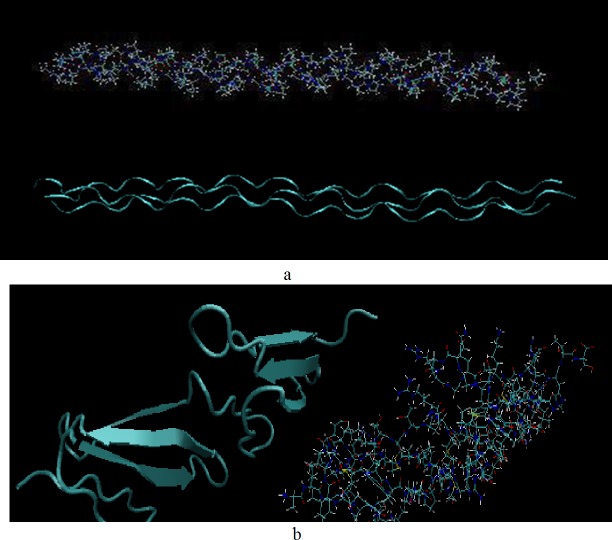
Molecular structures of the triple-helix collagen model (a), and fibronectin and its secondary structure (b). Arrows indicate anti-paralleled β-sheets in the fibronectin structure.

Collagen is the most abundant protein in the body, forming a considerable percent of muscles and tissues [29]. Furthermore, collagen is a crucial part of the ECM and necessary for tissue regeneration [30]. The initial atomistic structure of collagen (type I) is a triple-helix molecule with the diameter of 17.0 Å and the length of 116 Å (PDB, 1CLG) [31]. The structure is consisted of 108 amino acids (36 per each helix, Fig 1A) and is less computationally expensive than the other type of collagens, consisted of only repeating units of GLY-PRO-PRO with an ideal structure.

#### Fibronectin

Fibronectin is a model ECM protein which is crucially vital for cell adhesion and attachment [32–34]. In this study, a fibronectin module with a structure composed only of 3 antiparallel β-sheets connected through amino acids is selected as a representative model of fibronectin (PDB, 1FBR.pdb) [31]. This structure is composed of 93 amino acids as shown in Fig 1B, where the anti-parallel β-sheets represent unlike hydrophobicity. The structure has been previously employed in other studies connected with protein adsorption mechanism, leading to reliable results in good accordance with experimental observations [14,15,17]

#### Relaxation of protein structures

Isolated structures of the proteins are completely energy minimized after adding the hydrogen atoms in the calculated positions. The optimization procedure is then performed until an energy gradient lower than 10^−3^ kJ. mol^-1^. Å^-1^ is achieved. Unless otherwise stated, the optimization approach in this study is based on Smart algorithm which is in fact a cascade of some optimization methods including steepest descent, adopted basis Newton–Raphson, and quasi-Newton.

#### Modeling and relaxation of units of polymeric surfaces

**Fig 2:**
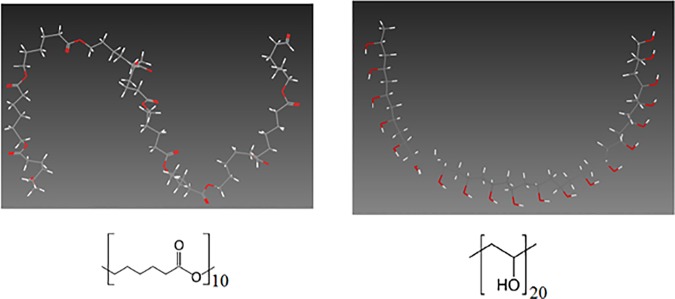
Molecular structure of a PCL oligomer (left) and a PVA oligomer (right) composed of 10 and 20 repeating monomers, respectively.

Oligomers of PCL and PVA with 10 and 20 repeating monomers, respectively, are generated in which the PVA oligomer is isotactic with the presence of some defects, as racemic dyads, along the main polymer chain. (Fig 2). Various ratios of PVA/PCL are obtained by combining different numbers of these oligomers, while keeping the total mass of the polymeric models constant. The mass ratios of PCL/ PVA biomaterials include 0:100 (pure PVA), 10:90, 30:70, 50:50, 70:30, and 100:0 (pure PCL). To keep the total mass constant, required number of each oligomer is calculated from Eqs (5) and (6) as:

$$n_{PCL}\times M_{PCL}+n_{PVA}\times M_{PVA}=V_{box}\times \rho_{box}$$ (5)

$$\frac{n_{PCL}}{n_{PVA}}\times \frac{M_{PCL}}{M_{PVA}}=mass\;ratio=\frac{m_{PCL}}{m_{PVA}}$$ (6)

where *n*_*PCL*_ and *n*_*PVA*_ denote the number of PCL and PVA oligomers, respectively. *M*_*PCL*_ and *M*_*PVA*_ also represent the molar mass of one oligomer of PCL and PVA, equivalent to 1143.46 g.mol^-1^ and 885 g.mol^-1^, respectively. In Eq (5), parameter *V*_*box*_ is also selected equivalent to 46.58 Å × 46.58 Å × 20 Å. It must be pointed out that the obtained values for number of oligomers are not integers and have been rounded to the best values that result in the desired mass ratio, but also minimizing the difference between actual and theoretical values of total mass.

To generate composite units of PVA/PCL surfaces in the MS software, Amorphous Cell module is employed to load different numbers of PVA and PCL oligomers into the system. The initial conformations of composite units of PVA/PCL systems are then obtained at 300 K with the specifications provided in Table 1. Parameter *ρ*_*box*_ is initially selected equal to 1.1 g/cm^-3^, similar to approximate density of bulk PVA and PCL, but right after generating different blends, NPT simulation at 300 K and 1 atm with a time step of 1 fs is performed for 150 ps to allow the systems find their realistic dimensions with regard to appropriate values of density.

**Table 1:** Properties used for generation of one unit and bulk models of the polymeric surfaces.

Sample code			One unit models		Bulk models	
	PCL (w %)	PVA (w %)	n_pcl_	n_pva_	n_pcl_	n_pva_
**PCL0**	0	100	0	33	0	297
**PCL10**	10	90	3	29	27	261
**PCL30**	30	70	8	23	72	207
**PCL50**	50	50	13	16	117	144
**PCL70**	70	30	18	10	162	90
**PCL100**	100	0	25	0	225	0
	Cell dimension		46.58 Å × 46.58 Å × 20 Å		141.33 Å × 141.33 Å × 20 Å	

The Amorphous module works based on finding all possible conformations of oligomers in a periodic cell. Nevertheless, its algorithm can be trapped in local minimum, thus leading to unrealistic results. To overcome this issue, a serious of MD simulations based on simulated annealing [35] are performed on the polymeric systems to provide a completely random redistribution of the polymeric chains. Table 2 represent the steps regarded in the simulations for equilibrium of the unit surfaces, followed by an additional geometry optimization using fine quality where an energy gradient lower than 0.005 kcal. mol^-1^. Å^-1^ is the criterion for complete convergence.

**Table 2:** Steps considered for equilibrium of unit surfaces of polymers cells.

Ensemble	Duration (ps)	Temperature (K)
NVT (time step = 0.1 fs)	10	310
NPT (P = 1atm)	150	310
NVT	200	400
NVT	200	550
NVT	150	300
NVT	200	750
NVT	350	1000
NVT	200	850
NVT	150	600
NVT	200	450
NVT	250	310

#### Relaxation and generation of bulk polymeric surfaces

**Fig 3:**
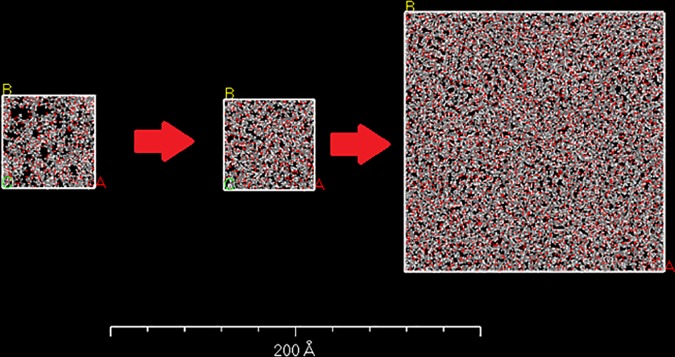
In-cell representation of the unit and bulk models of the polymeric surfaces before and after performed simulations for equilibrium.

**Fig 4:**
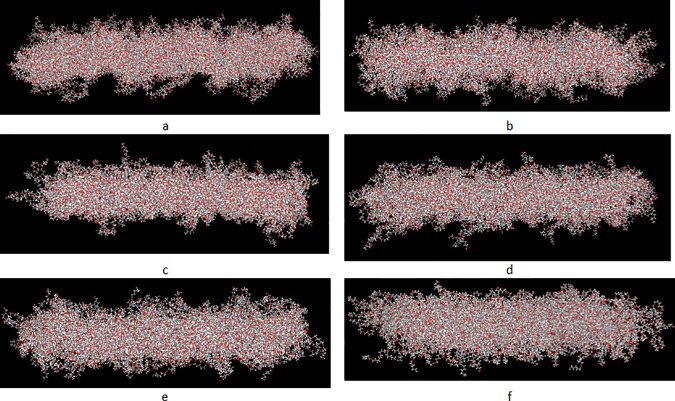
Generated bulk models using different blends of PCL/PVA oligomers. a) PCL0,b)PCL10, c)PCL30,d)PCL50, e)PCL70, and f) PCL100.

After relaxation of the unit surfaces of the polymer systems, the bulk models are obtained by replicating the unit surface, 3 times in the x-y plane perpendicular to the coordinate of the simulation cell thickness. Subsequently, a number of MD simulations as indicated in Table 3 are performed on the bulk models to help to redistribution of the polymer chains within the system. The protocol for relaxation of bulk models is based on the methodology explained in [36,37] for obtaining equilibrium state of a similar bulk polymer cell. Initial and final structures of a sample polymer model is depicted in Fig 3. Additionally, Fig 4 indicates the generated bulk polymer using the mentioned method.

**Table 3:** Steps considered for equilibrium of bulk surfaces of polymers cells.

Ensemble	Duration (ps)	Temperature (K)
NVT (time step = 0.1 fs)	50	310
NVT	250	600
NVT	400	310
NPT (P = 1atm)	350	310
NVT	400	750
NVT	500	1000
NVT	450	1400
NVT	450	750
NVT	250	500
NVT	400	310

#### Calculation of adhesion energy

**Fig 5:**
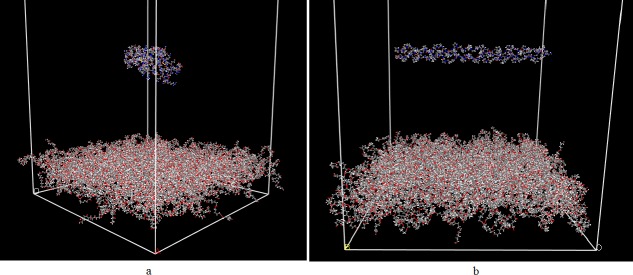
Initial configuration of layers. a) collagen, and b) fibronectin.

To generate final protein-polymer systems, first, the proteins are placed in vicinity of the bulk polymer systems. The simulation cell is then extended as long as 300 Å along the polymer thickness coordinate, to prevent interactions with the above fake layer (Fig 5). The protein structures are then geometry optimized in an effective dielectric medium mimicking water, achieved by considering the distance-dependent dielectric constant equal to that of water as also previously employed in other studies [10,12,13,15]. The simulations are ultimately performed for 2.5 ns, with time step of 1 fs, using NVT ensemble based on Berendsen thermostat keeping the temperature constant at 310 K. The cut-off distances for van der Waals and hydrogen bond interactions are selected 12.5 Å, and 4.5 Å, while Ewald-based electrostatic interactions are considered with accuracy of 0.001 kcal/mol. During the simulations, the polymer atoms are completely fixed while protein atoms are completely free.

To assess whether the final system has reached the steady state condition, fluctuations around the value of 3*k*_*b*_TN/2 were measured, where *T* is the temperature, *N* denotes total number of molecules, and *k*_*b*_ is Boltzmann constant. It was observed that after the specified simulation time, all systems were able to reach steady state conditions. Subsequently, the adhesion energy was calculated from the following formulation:

$$E_{adhesion}=(E_{protein}+E_{biomaterial})-E_{total}$$ (7)

where *E*_*protein*_ and *E*_*biomaterial*_ denote the energy of protein and polymeric biomaterial in the system, respectively, and *E*_*total*_ is the total energy of the system after equilibrium. Because the polymer models are completely fixed, the value of *E*_*biomaterial*_ is conveniently equal to zero. Based on this formulation, a large positive value for *E*_*adhesion*_ represents strong adhesion to the biomaterial surface, while a small positive value or a negative value indicates weak adhesion.

### Experimental procedure

#### Electrospinning process

Hybrid scaffolds composed of PCL and PVA nanofibers are manufactured using co-electrospinning method. In this regard, a 10% w/v solution of PCL (Mn = 80 kDa, Sigma-Aldrich) is prepared by dissolving the polymer in a 1:1 solution of chloroform and methanol (Merck) stirred for 3 hours at room temperature. Moreover, PVA (Mn = 72 kDa, Merck) is dissolved in deionized (DI) water and stirred for 2 hours at 80°C, to obtain a homogenous solution. Simultaneous co-electrospinning of PCL and PVA solutions is then performed with various flow rates, to obtain different composite structures. Accordingly, pure PCL and PVA scaffolds, along with PVA/PCL electrospun scaffolds including: 30% (PCL30), 50% (PCL50), 70% (PCL70) of PCL are fabricated. All experiments are performed with four times replications, unless otherwise stated.

#### Fiber characterization

The morphological characteristics of nanofibers are investigated via scanning electron microscopy (SEM, XL30 model, Philips) at voltage of 20 KV. The hydrophilicity of surfaces is also characterized using drop shape analyzer. Photographs of distilled water drop are taken by camera (AM-4113ZT4, DinoLite), and, water contact angles are analyzed using DinoCaoture.

#### MTT cell viability assay

It is widely believed that due to excellent biocompatibility, composite PCL/PVA electrospun scaffolds can be used for various tissue engineering and drug delivery applications. In most functions, in addition to the specific cell types of the damaged tissue, endothelial cells should have the ability to proliferate in the damaged zone to facilitate the angiogenesis process, which is one of the key factors in tissue regeneration [38,39]. In this regard, human endothelial cells (HEC) are selected to be cultured on the scaffolds during MTT cell viability assay. HUVEC (a cell line drived from Human Umbilical Vein Endothelial Cells) is purchased from Pasteur Institute of Iran.

To this aim, HEC are first cultured in Dulbecco’s modified Eagle’s medium (DMEM), supplemented with 10% of fetal bovine serum (FBS) and 1% of penicillin/streptomycin. Subsequently, the cells are incubated at 37°C and 5% CO_2_ and detached by trypsin-EDTA at the confluence of 80%. For the MTT assay, the cells are cultured on the scaffolds with the area of 1.9 *cm*^2^ placed in 24-well plates. The wells coated with polyester are considered as the control group. MTT [3-(4,5-Dimethylthiazole-2-yl)-2,5-diphenyltetrazolium] assay is carried out to quantify the cell viability. Accordingly, 5 mg of MTT (Sigma) is dissolved in 1 ml of phosphate-buffered saline (PBS) solution and sterilized by filtering. The culture medium of the 24 well plates is then replaced with 50 μl of MTT solution and 500 μl of fresh culture medium, and incubated to form formazan crystals by mitochondrial dehydrogenases. After 4 hours, the medium is removed, and 200 μl Dimethyl sulfoxide (DMSO) is added to each well to dissolve the formazan crystals. The optical density of the solution is finally obtained using an Elisa plate reader at the wavelength of 570 nm.

## Results

### Adhesion energy

**Fig 6:**
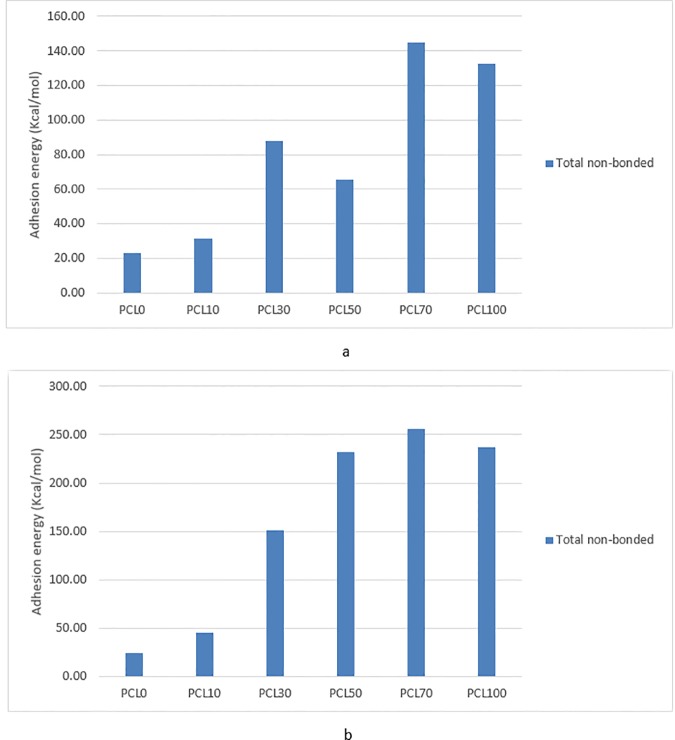
Mean of adhesion energy for a) collagen, b) fibronectin during the simulations.

Fig 6. indicates the mean of adhesion energy in the last 250 ps of simulations obtained from MD simulations of all 12 systems after 2.5 ns. Results show that, in the first place, the protein adsorption is highly dependent on the composition of the substrates such that an increase in the percentage of PCL generally leads to stronger adsorption for both proteins. It is also deduced that the adsorption mechanism for two protein fragments is more dominantly affected by the surface composition rather than the protein structure. Accordingly, adhesion energy for both fragments lies in a similar range, however, the collagen structure generally represents stronger adsorption energy compared with fibronectin. Furthermore, considering both protein fragments, the best protein adsorption is similarly achieved in sample PCL70.

A sharp rise in the adhesion energy of collagen is seen from sample PCL10 to PCL30, resulting in an approximate increase, as much as 1.5 times, in adhesion energy. This finding indicates that the scaffolds with more than 30% of PCL are expected to be more desirable for adsorption of collagen from a theoretical point of view in molecular scale. In case of fibronectin, the same substantial increase in adhesion energy is observed from sample PCL50 to PCL 70, making the energy of PCL70 almost 1.8 times higher than PCL50. Therefore, a suitable scaffold from a theoretical point of view based on best fibronectin adsorption can be expected to be in the scaffolds with more than 70% of PCL.

In addition to physical and chemical characteristics of the substrates, hydrophobicity of protein residues is another important factor in the interactions of protein and surfaces [13,15,40]. The present collagen structure is composed only of GLY and PRO amino acids, which both are relatively hydrophobic residues. Therefore, increasing the hydrophobicity of substrate is expected to be in favor of adhesion energy, which is also more apparent in simulation results of collagen. However, in case of collagen, the adhesion energy for samples with 30 or more percentages of PCL are observed to represent no significant rise in adhesion energy, showing that collagen adsorption almost reaches a plateau in PCL50.

Considering fibronectin, the structure in this study is primarily composed of β sheets with more varied electric charge and hydrophobicity. Therefore, more favorable protein adsorption is only achieved when the surface is largely composed of PCL (PCL70 and PCL100), making fibronectin less sensitive to the variations of the surface chemical composition.

### Final protein conformation

**Fig 7:**
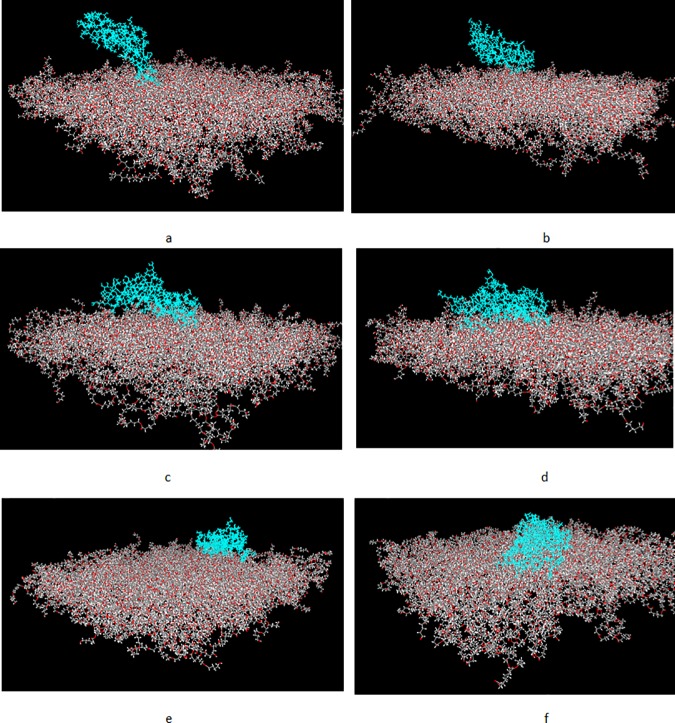
Final conformations of fibronectin structure after 2.5 ns relative to different blends of PCL/PVA. a) PCL0,b)PCL10, c)PCL30,d)PCL50, e)PCL70, and f) PCL100.

**Fig 8:**
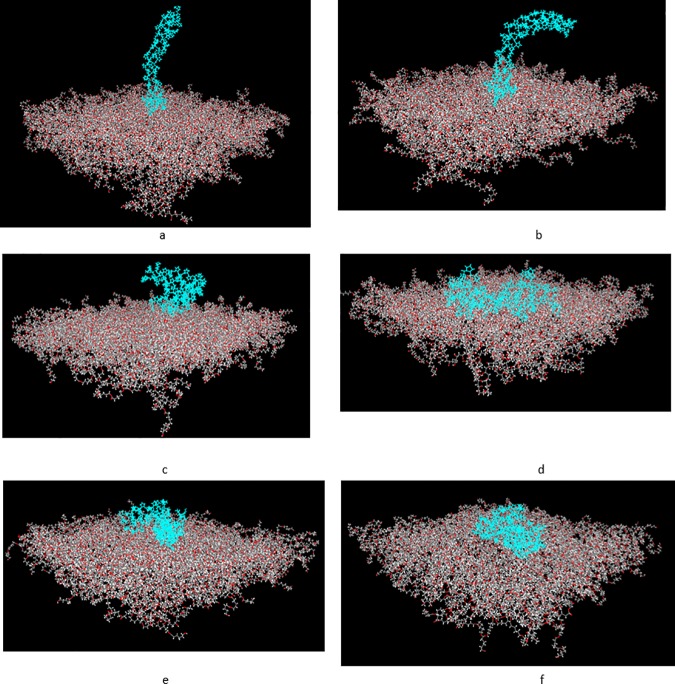
Final conformations of collagen structure after 2.5 ns relative to different blends of PCL/PVA. a) PCL0,b)PCL10, c)PCL30,d)PCL50, e)PCL70, and f) PCL100.

Final confirmations of proteins at the end of simulations are shown in Figs 7 and 8 for fibronectin and collagen, respectively. Although due to time and size limitations, these simulations only provide approximate conformations, study on the final rearrangements and structural deformations of proteins can provide a more precise insight into understanding the mechanism of protein adsorption to the polymeric surfaces. It is well documented in the literature that conformational rearrangements are associated with stronger protein adsorption [12,15]. Accordingly, both proteins have undergone significant conformational rearrangements, specifically, structural denaturation in case of collagen can be observed, in particular, relative to PCL 50–100 (Figs 7 and 8).

Furthermore, it can be observed that, the increase in the percentage of PCL in the surfaces is accompanied by an increase in the number of interacting residues with the surfaces in a way that a larger number of residues in the protein structure comes into contact with the polymeric surfaces. Notably, in case of collagen relative to PCL0 and PCL10, the protein structure lies relatively horizontal in regard to the surface, while retaining its initial secondary structure. However, in the samples with more enhanced hydrophobicity, such as PCL70 and PCL100, the protein structure spreads on the surface, losing initial secondary structure composed of alfa-helices.

In case of fibronectin, the protein structure ultimately comes into contact with all polymeric surfaces, yet it represents a pronounced tendency to attach to the more hydrophobic surfaces such as PCL 50–100. In line with collagen, the fibronectin structure also represents large structural deformations to optimize residue-surface interactions.

### Fiber Characterization

**Fig 9:**
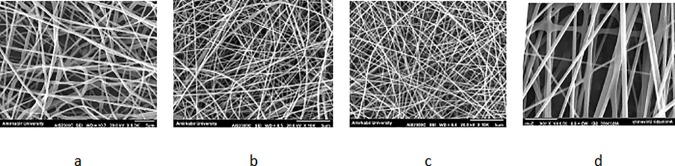
SEM photographs of PCL and PVA with different flow rate. a) PCL = 1 cc/h, b)PVA = 1cc/h, c)PVA = 0.43 cc/h, and d)PVA = 2.3 cc/h.

**Fig 10:**
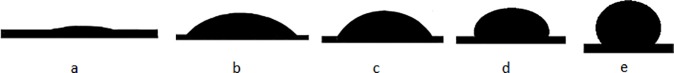
Water contact angle measurement of the electrospun scaffolds. a) PVA = 0°, b)PCL30 = 44°, c) PCL50 = 59°, d)PCL70 = 98°, and e) PCL100 = 131°.

Electrospun scaffolds composed of PCL and PVA nanofibers with various compositions are manufactured according to Table 4. Additionally, Fig 9 demonstrates the SEM images of nanofibers, proving the capability of the employed electrospinning procedure to prepare the nanofibers without any beads. The hydrophobicity of the electrospun substrates is also determined using drop shape analyzer. It is concluded that as the percentage of PCL increases, the surfaces become more hydrophobic (Fig 10), therefore, the hydrophobicity is controlled through the surfaces. In this regard, the pure PVA sample has the lowest water contact angle due to extreme hydrophilic characteristics of PVA, while PCL represents the largest water contact angle due to the hydrophobic nature of PCL.

**Table 4:** Flow rates of PCL and PVA for various compositions.

	Flow rate (cc/h)	
Scaffold code	PCL	PVA
**PCL0 (pure PVA)**	0	1
**PCL30**	1	2.3
**PCL50**	1	1
**PCL70**	1	0.43
**PCL100 (pure PCL)**	1	0

### MTT assay

**Fig 11:**
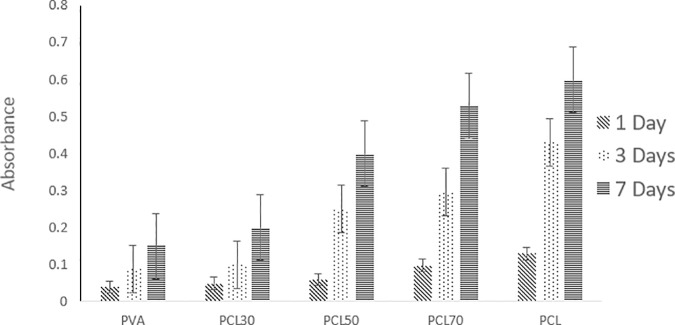
MTT assay for endothelial cells during one week.

MTT cell viability assay is performed to evaluate the attachment and proliferation of endothelial cells on the electrospun scaffolds (Fig 11). As described earlier, the scaffolds show different capability in cell proliferation as the percentage of PCL and PVA is modified. The results at day 1 indicate that the samples containing 70 and 100 percentages of PCL have the highest biocompatibility and initial cell attachment, approximately 3 times greater than the PVA nanofibers. The results at days 3 and 7 also indicate an increase in cell proliferation capability during one week. Moreover, while PVA and PCL30 have low biocompatibility, other samples show significant rise in cell proliferation during this period of time. It can therefore be concluded that according to the performed MTT assay, the samples with more than 50% of PCL, in particular, PCL70 and PCL100 are more favorable for initial cell attachment and also eventual cell proliferation, hence, more desirable for tissue engineering purposes.

## Discussion

In this study, a theoretical approach was coupled to experimental assays to obtain the most biocompatible designs of a composite electrospun scaffold. To this aim, first, a computational approach based on MD simulations was used to investigate the adsorption mechanism of two ECM proteins, with similar size but unlike secondary structure, fibronectin and collagen, on heterogeneous surfaces composed of two prevalent polymers in tissue engineering and drug delivery with completely unlike hydrophobicity, namely, PCL and PVA. The hydrophobicity of PVA/PCL models was modified through loading different numbers of oligomers into the system, while keeping the overall mass of the polymeric models constant. Subsequently, interactions of different blends of PCL/PVA with fibronectin and collagen were modeled, and the adhesion energy along with final conformation of proteins were studied. Next, MTT assay was also performed on electrospun PCL/PVA scaffolds, with similar compositions to MD models, to evaluate cell proliferation on the scaffolds and investigate the possibility of using MD simulation results along with experimental findings for obtaining optimized polymeric scaffolds with best biocompatibility in terms of both protein adsorption and cell attachment. This therefore can alleviate the effort required for finding the best polymeric compositions by extensive experimental assays *in vitro*, since early MD results of ECM protein adsorption can provide a reasonable estimate of the performance of different compositions of scaffolds before experimental characterizations.

In view of the computational part, MD simulations indicated that the hydrophobicity of the substrate and protein residues as well as the size of protein had significant effects on adhesion energy. Accordingly, a similar trend in adhesion energy was observed for both fibronectin and collagen, i. e. increasing the percentage of PCL in the PVA/PCL surfaces generally led to stronger protein adsorption. This increase in the adhesion energy was attributed to an increase in the hydrophobicity of the surface accompanied by increasing the percentage of PCL. This observation was in good agreement with previous theoretical [15,17] and experimental [41,42] research studies of other groups reporting better protein adsorption was achieved by increasing the surface hydrophobicity. In this study, it was also found that the substrate composition, in comparison to other factors such as the structure, and hydrophobicity of the proteins, played a more dominant role in the strength of protein-biomaterials interactions. Moreover, two significant increases in the adsorption energy were observed by increasing the percentage of PCL from 10% to 30% for collagen, and from 50% to 70% for fibronectin. However, adsorption of both protein fragments, in particular collagen, was accompanied by reaching a plateau after which the adsorption either did not improve significantly or deteriorated. Unexpectedly, the most hydrophobic surface was not the best sample for protein adsorption.

A comparative study on the final conformation of proteins demonstrated that all proteins exhibited large conformational rearrangement considering all samples of polymeric surfaces, in particular, the more hydrophobic surfaces with more than 50% of PCL. Moreover, the secondary structure in collagen was significantly lost at the end of the simulations in regard to extremely hydrophobic surfaces.

Considering the experimental part of this study, based on co-electrospinning of PVA, and PCL solutions in various ratios, it was indicated that surface hydrophobicity had great effects on the scaffolds biocompatibility, since the surface with higher hydrophobicity represented a better substrate for a more pronounced cell adhesion and proliferation. The electrospun samples PVA and PCL30 demonstrated poor cell proliferation, while a significant rise was observed in cell proliferation for the rest of the samples, in particular, PCL70 and PCL100, during one week.

These experimental findings were in good agreement with theoretical point of view obtained from MD results indicating that the samples containing more than 70% of PCL (considering both collagen and fibronectin simultaneously) had a stronger capability for protein adsorption. By comparing both experimental and theoretical views, it is concluded that MD simulations can potentially provide a good initial estimate for cell attachment an eventual cell proliferation based on the adhesion of fibronectin and collagen, two ECM proteins, on the surface. Therefore, it is desirable to eliminate the samples with less than 50% of PCL and consider only the remaining samples for future in vitro, in vivo evaluations.

This study proves that a computational approach based on MD simulations can be considered as a powerful asset for better understanding of the tendency of protein adsorption on different surface compositions, thus optimizing the biomaterials performance. Although the results of MD simulations are based on an extremely limited timeframe and dimension, as restricted as nanoscales, it can still shed light on the nature of interactions between different surfaces and ECM proteins. In this study, MD results indicate that based on adsorption of collagen and fibronectin protein fragments, the best composition of a PCL/PVA surface is likely to be achieved in more than 50% of PCL, in particular 70% and 100% of PCL. However, it is worth-mentioning that experimental results indicate that the best biocompatibility is achieved in 100% of PCL which is in contrast to simulation results, introducing the sample with 70% of PCL as the most desirable one.

According to this study, the samples with higher adhesion of fibronectin and collagen support a higher degree of cell viability. These findings are in good agreement with previous research studies, where stronger ECM protein adsorption is found to be in harmony with better cell proliferation [4–6]. More specifically, adsorption of collagen in this study offers a wider range of samples for optimization considerations, unlike the fibronectin results that restrict the optimum samples only to the last two ones (PCL70 and PCL100).

The direct correlations between adsorption of ECM proteins and biocompatibility of scaffolds have been previously reported based on experimental studies, in particular the important role of endothelial cells in biocompatibility [43,44]. However, in this study, a parallel computational-experimental approach is employed to address the influence of surface composition of a heterogeneous amorphous biomaterial on its protein adsorption. Despite significant importance of design of optimum polymeric scaffolds in tissue engineering, only a limited number of parallel experimental-computational studies exist in the literature concerning optimizing the biocompatibility of scaffolds based on protein adsorption. One such study deals with study on the improvement of fibronectin adsorption to different functionalized PCL surfaces [16].

Hence, MD simulations can be considered as a powerful asset to optimize the percentages of polymers in amorphous scaffolds, reducing the effort required for time-consuming and expensive experiments on all samples. Results of this study highlight the role of MD simulations in obtaining optimized scaffolds with better biocompatibility, as protein adsorption phenomenon cannot be captured using continuum formulations and coarse-grained modeling. This study is the first to address application of MD simulation in optimization of an amorphous polymeric scaffold. Therefore, parallel computational-experimental optimization approaches can contribute to a significant understanding of the interplay between protein and surface interaction. Future works in this regard can be parallel computational-experimental studies on optimization of nanostructured biomaterials such as carbon nanotube-polymeric composites and amorphous structures with more complex types of polymers. A comparison can also be performed between results of simulations and other type of polymeric scaffolds such as hydrogels or sponge-like biomaterials.

## Conclusions

In this study, a computational approach linked with experimental assays was employed to optimize biocompatibility of a composite PCL/PVA scaffold. The coupling was based on the correlation between stronger ECM proteins adsorption and enhanced cell proliferation on a biomaterial. In this regard, six different amorphous surfaces with different percentages of PCL and PVA were considered. Results of computational approach obtained from MD simulation of fibronectin and collagen adsorption indicated better adsorption for samples with more than 50% of PCL, in particular 70% and 100% of PCL. The best protein adsorption regarding both models was achieved in a surface with 70% of PCL. Cell viability assay using MTT assay also represented samples with 70% and 100% of PCL provided the best biocompatibility. In particular, the optimum cell proliferation was observed for the sample with 100% of PCL. A comprehensive study was also done on the final conformation of proteins relative to different surfaces. Results of this study introduced MD simulation as a powerful tool to optimize the composite of new structures based on their ECM protein adsorption capability. Therefore, based on the simulation results, the burden of large number of experiments required for a diverse number of compositions can be alleviated by narrowing down the samples to those with the best ECM protein adsorption baesd on simulation results. These findings are beneficial for utilization of composite PCL/PVA as nanofibers or drug carriers in tissue engineering and biomedicine.

## Supporting Information

S1 File Modified Forcefield file. (OFF)

S1 Table Data of adhesion energy calculation. (XLSX)
